# Editorial for the Special Issue “COVID-19 Vaccines: A Public Health Perspective”

**DOI:** 10.3390/vaccines11081379

**Published:** 2023-08-18

**Authors:** Chiara de Waure, Chiara Cadeddu, Aldo Rosano

**Affiliations:** 1 Department of Medicine and Surgery, University of Perugia, 06132 Perugia, Italy; chiara.dewaure@unipg.it; 2 Section of Hygiene, Department of Life Sciences and Public Health, Università Cattolica del Sacro Cuore, 00168 Rome, Italy; 3 National Institute for the Analysis of Public Policy—INAPP, 00198 Rome, Italy; aldo.rosano@iss.it

Public health is aimed at protecting and promoting citizens’ and communities’ health through different interventions, including vaccinations. The COVID-19 pandemic has emphasized the importance of public health interventions to counter the consequences of the rapid spread of SARS-CoV-2 among the worldwide population. 

COVID-19 vaccines have been among the last measures adopted to protect people and communities from the consequences of the pandemic, but they have also been the most successful. 

Nonetheless, the planning and implementation of a vaccination campaign requires considering several aspects that go beyond the vaccines’ specific characteristics, such as their characteristics, availability, safety and efficacy [1]. In fact, health systems’ capacity to manage and organize the vaccination campaign together with the public response are undoubtedly relevant aspects for guaranteeing a successful vaccination campaign [1].

The purpose of this Special Issue was to gather articles addressing these topics and convey the relevant and useful information for public health decision makers. The Special Issue collected seven contributions from different settings and perspectives, some of which have an international scope. 

The two main topics addressed by the articles of this Special Issue were the organizational/logistical aspects of the COVID-19 vaccination campaign and the hesitancy towards vaccination and the reasons behind it. 

In terms of the organizational/logistical aspects, the articles collected in this Special Issue highlighted that differences existed across countries in the planning, organization, and implementation of the COVID-19 vaccination campaigns that had impacts on the speed of achievement of vaccination coverage goals. A very relevant aspect that was also addressed was represented by the settings in which vaccination was offered, with a focus on the role of mass vaccination centers. These centers have been already used for other vaccination campaigns and have the advantage in optimizing, in terms of resources used and time, vaccination deployment. Nevertheless, attention should be paid to the internal organization of these centers as well as to identification, recruitment, and training of the necessary health professionals. 

Health professionals are a key contributor to the success of a vaccination campaign, including the COVID-19 one for the following reasons: they are the most used source of information by the general population [2,3], they are actively involved in administering vaccines and they are also targets of the vaccination campaign.

Nevertheless, the attitudes and behaviors towards vaccination of the population also depend on several other aspects that have been addressed by the articles included in this Special Issue. Among them, public opinions and sentiments have been also investigated as an important element contributing to the public response to COVID-19 vaccination.

Eventually, the role of the timely collection and analysis of data, such as those on breakthrough infections, was also emphasized as the first milestone in recognizing a potential concern, hypothesizing potential causes and identifying possible solutions in what should be a virtuous circle of actions that should be implemented for evidence-based health planning. 

The main topics addressed in this Special Issue—organization of the vaccination campaign and hesitancy—had substantially different degrees of significance at different stages of the epidemic. In the first months of vaccine deployment, organizational issues were more relevant and impactful, while vaccine hesitancy was rather low. Conversely, in the later stages of the pandemic, the organizational aspects were mostly fixed but there was the issue of coping with increasing vaccine hesitancy in the face the need to administer booster doses because of the natural waning of immune protection from the vaccines and the emergence of new variants of SARS-CoV-2.

Undoubtedly, the lessons learned from the COVID-19 pandemic in terms of hesitancy is peculiar and cannot be widely applied to other vaccinations. However, it pointed out several aspects that should be thoroughly considered for the preparedness for future pandemics, like the importance of a clear communication, including the utilization of new media, and the relevance of trust in the scientific community and institutions. On the other hand, from the organizational point of view, the pandemic has reiterated the importance of health service research to understand the role of structures and processes in delivering high-quality healthcare services. Understanding these elements, beyond being useful for health system preparedness, could also advance other vaccination programs. A crucial issue in this respect is the transferability of organizational models: the best practices in COVID-19 vaccination delivery could be studied in terms of adaptability and scale up to be extended to other settings but also other vaccinations.

Future studies in these research areas, if conducted in different countries and settings, could allow comparative analyses that facilitate the recognition of best practices and the improvement of public health around the world. 
